# U-shape rotating anti-cathode compact X-ray generator: 20 times stronger than the commercially available X-ray source

**DOI:** 10.1107/S0909049513022188

**Published:** 2013-10-02

**Authors:** N. Sakabe, K. Sakabe, S. Ohsawa, T. Sakai, H. Kobayakawa, T. Sugimura, M. Ikeda, M. Tawada, N. Watanabe, K. Sasaki, M. Wakatsuki

**Affiliations:** a Photon Factory, High Energy Accelerator Research Organization (KEK), 1-1 Oho, Tsukuba 305-0801, Japan; b Foundation for Advancement of International Science (FAIS), Kasuga 3-chome, Tsukuba, Ibaraki 305-0821, Japan; c Accelerator Laboratory, High Energy Accelerator Research Organization (KEK), 1-1 Oho, Tsukuba, Ibaraki 305-0801, Japan; d Synchrotron Radiation Research Center, Nagoya University, Chikusa, Nagoya, Aichi 464-8603, Japan; e Nagoya University, Chikusa, Nagoya, Aichi 464-8603, Japan; f National Institute of Advanced Industrial Science and Technology (AIST), 1-1-1 Higashi, Tsukuba, Ibaraki 305-8568, Japan

**Keywords:** X-ray generator, high-brightness X-ray generator, compact X-ray generator, U-shape anti-cathode in X-ray generator

## Abstract

A new type of U-shape anti-cathode X-ray generator in which the inner surface of a cylindrical target is irradiated by an electron beam has been made by modifying a conventional rotating anti-cathode X-ray generator whose brightness in the catalog is 12 kW mm^−2^. The target material (Cu), target radius (50 mm) and rotating speed (6000 r.p.m.) were not changed in this modification. A brightness of 52 kW mm^−2^ was obtained by this U-shape-type X-ray generator. This means that the brightness of the new type is 4.3 times greater than that of the old unmodified one. Furthermore, the new-type X-ray generator yielded a brightness of 129 kW mm^−2^ by adding a carbon coating on the Cu target. This means an overall increase of brightness of ten times. The original generator has the highest brightness in the generators of the same class (having a radius of 50 mm and rotation speed of 6000 r.p.m.). Observations showed that Cu *K*α counts at vertical incidence of the electron beam onto the surface of the new target, which is initially optically smooth, decrease as the surface is roughened by a severe thermal stress caused by strong electron beam exposure. Further observation reveals, however, that oblique incidence of the electron beam onto the roughened surface drastically increased the X-ray output and amounts to twice as much as that from a smooth surface at vertical incidence. Thus, at the present stage, an overall increase of brightness has been realised at a level 20 times stronger than that of the original commercially offered X-ray generator that we modified.

## Abstract 2

A new type of U-shape anti-cathode X-ray generator in which the inner surface of a cylindrical target is irradiated by an electron beam has been made by modifying a conventional rotating anti-cathode X-ray generator whose brightness in the catalog is 12 kW mm^−2^. A brightness of 129 kW mm^−2^ was thereby obtained with this new U-shape-type X-ray generator. This new X-ray generator is expected to be of keen interest for applications in academia, industry and in hospitals.

## 1. Introduction

Conventional rotating anti-cathode X-ray generators are used not only for academic research laboratories but also in industry and in hospitals where sometimes they also need high-brightness X-ray beams. Conventional commercial rotating anti-cathode X-ray generators, however, have fundamental limitations such as an electron beam power density that cannot exceed the level corresponding to melting of the irradiated target as well as the electric discharge limit and the surface damage of the target caused by thermal stresses. These factors are obstacles to increasing the X-ray brightness.

To overcome the limitations mentioned above, a new type of U-shape rotating anti-cathode (Sakabe, 2002 ▶) X-ray generator combined with an electron gun which has a focusing bending magnet (Ohsawa, 2008 ▶) was developed and a brightness of 52 kW mm^−2^ was achieved (Sakabe *et al.*, 2008 ▶; Sugimura *et al.*, 2008 ▶); building on this, a brightness of 128.7 kW mm^−2^ was achieved (Sakai *et al.*, 2011 ▶) by coating the target with a carbon film (Sakabe *et al.*, 2008 ▶). Many tiny grains on the target were observed (Sakabe *et al.*, 2008 ▶). These grains presumably absorb the X-rays extracted *via* the Be window outlet in a direction 6° from the target surface. Here we show how this problem was solved.

## 2. Outline description of the U-shaped Cu anti-cathode X-ray generator

The new-type U-shape anti-cathode X-ray generator in which the inner surface of the cylindrical target is irradiated by an electron beam (Sakabe *et al.*, 2008 ▶) was made by modifying a conventional rotating anti-cathode X-ray generator, type M06XHF22-Fine (made by MacScience when it was in business), and whose brightness cited in the commercial catalog is 12 kW mm^−2^. The target material (Cu), target radius (50 mm) and rotating-anode speed (6000 r.p.m.) were not changed in this modification.

**Figure 1:**
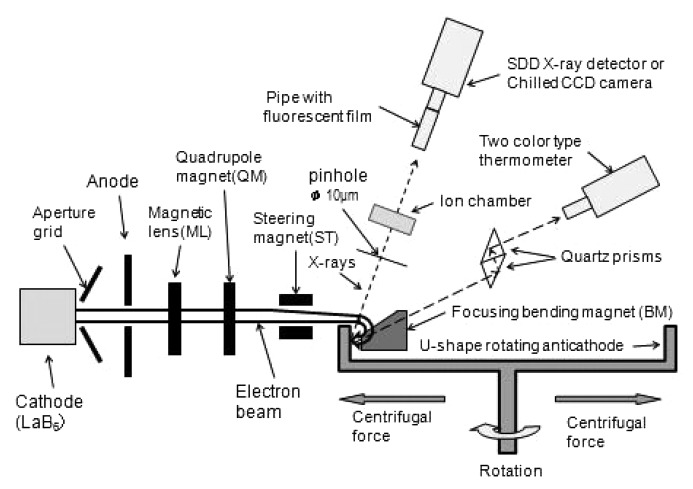
Schematic view of the X-ray generator with the U-shape rotating anti-cathode (the U-shape is the cross-section shape of a cylindrical annulus) and the arrangement of the detectors of different types and functions.

Thermal electrons from a hot LaB_6_ cathode on which a negative electric high voltage is supplied are extracted and accelerated by an aperture grid to which is applied a +3 kV higher voltage than the cathode, and which pass through a central hole of the grid electrode as shown in Fig. 1 ▶. The electron beam is further accelerated by the anode potential and passes through the central hole of the anode which has a ground potential.

Behind the anode, all parts are grounded; in other words, an electric potential difference does not exist anywhere. This means that, whatever the target temperature is increased to, an electric discharge does not occur in the whole area behind the anode position in Fig. 1 ▶.

The electrons of the beam are rarely captured by the aperture grid or the anode, and thus the anode temperature does not increase. The shape of the cross section and the direction of the electron beam are aligned by a magnetic lens, a quadrupole magnet and a steering magnet. Finally, the electron beam is deflected by about 180° by the focusing bending magnet (BM) and focused onto the U-shape rotating target.

Since the focal length of the BM can be designed to be only a few millimeters in length, the focal size of the electron beam is adjustable to about 40 µm on the target surface (Sugimura *et al.*, 2008 ▶). Since the electron beam direction is changed by 180° by the focusing bending magnet, even if the target metal were evaporated by the high temperature the possibility for resultant vaporized metal atoms hitting the cathode is very small.

The focusing BM has a gap of 8 mm between its north and south magnetic poles and the distance from the target is set to be 2 mm long. X-rays from the target are taken out through the gap between the magnetic poles and then extracted *via* a Be window. The take-off angle is 6°.

A quartz window is provided for a two-color-type thermometer (Thermera-seen D414) to measure the temperature on the target surface near the irradiated point. The observation angle from the target surface is 75°.

## 3. Experimental and results

### 3.1. First stage of the experiment

**Figure 2:**
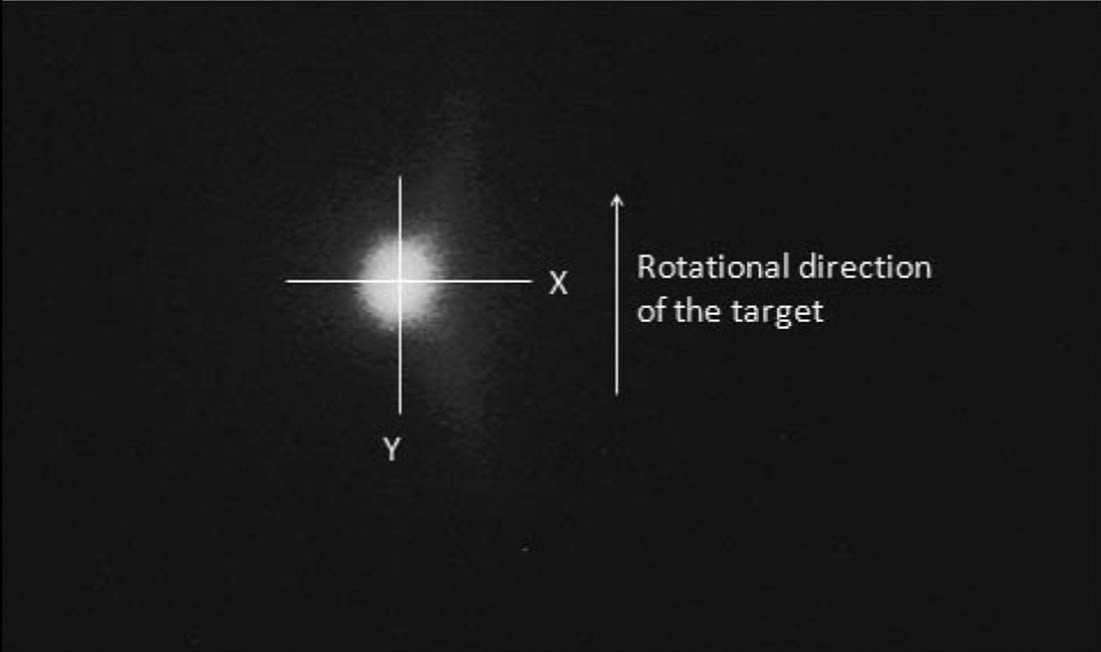
X-ray source image through a 10 µm-diameter pinhole.

The X-ray source, namely the electron beam focus, has a two-dimensional area. Its size along two orthogonal axes has been measured *via* an X-ray image taken by the pinhole camera method. A 10 µm-diameter pinhole was put at a distance of 75 mm from the irradiated point, behind which a CCD camera with a thin fluorescent film at its front-end was set as shown in Fig. 1 ▶. The distance between the pinhole and the fluorescent film was 750 mm, so that the image magnification at the film became 10×. This magnification factor was further confirmed by measuring the X-ray image displacement with a micrometer when the CCD camera was moved in a direction perpendicular to the optical axis. Fig. 2 ▶ shows the X-ray source image at a generator setting of 60 kV, 45 mA.

Since the temperature near the electron-beam-irradiated area of the target exceeded the melting point of the target metal Cu by a few hundred degrees, a coating treatment to suppress the evaporation of the target metal was required to further improve the brightness. An estimation of the efficiency of the coating methods was reported in a previous paper (Sakabe *et al.*, 2008 ▶), and on which this new paper and study builds. The pressure produced by a 1 µm depth of graphite film with an applied centrifugal force of 2000*g* is 44.4 Pa. On the other hand, the vapor pressure of Cu at 1700 K, which is 344 K higher than the melting point of Cu, is 16 Pa, sufficiently lower than the above-mentioned covering pressure.

### 3.2. Coating film on the target

**Figure 3:**
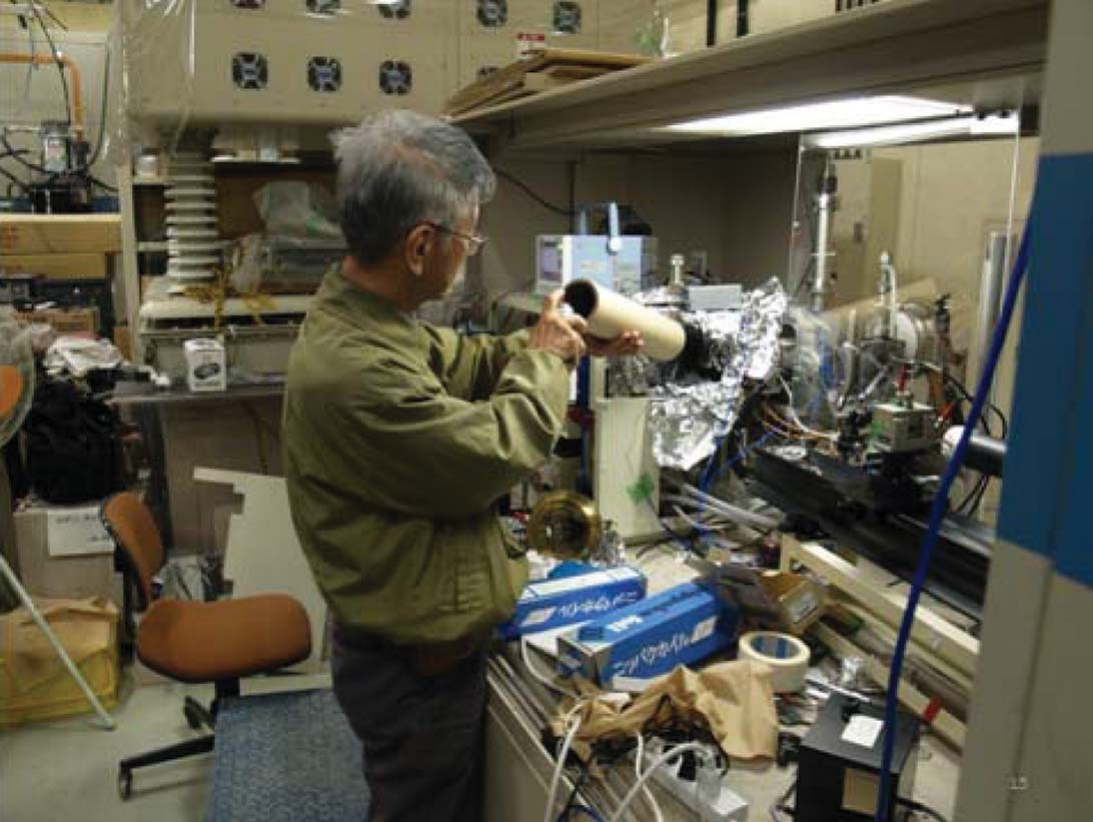
Target being coated with Aerodag G spray by N. Sakabe.

One of the best materials to coat a Cu target is graphite because its vapor pressure is very low. It has a density smaller than that of Cu and its reactivity and/or solubility to Cu is negligible. Furthermore, its electrical conductivity is adequate. Even if a dense coating film was made by a physical or chemical vapor deposition process, a thin film would develop cracks when the irradiated spot melted. To avoid cracks, it is better to coat the target by spraying with micro-particle graphite; to this end we used a spray bottle called ‘Aerodag G’ (manufactured by Henkel Corporation) (see Fig. 3 ▶).

**Figure 4:**
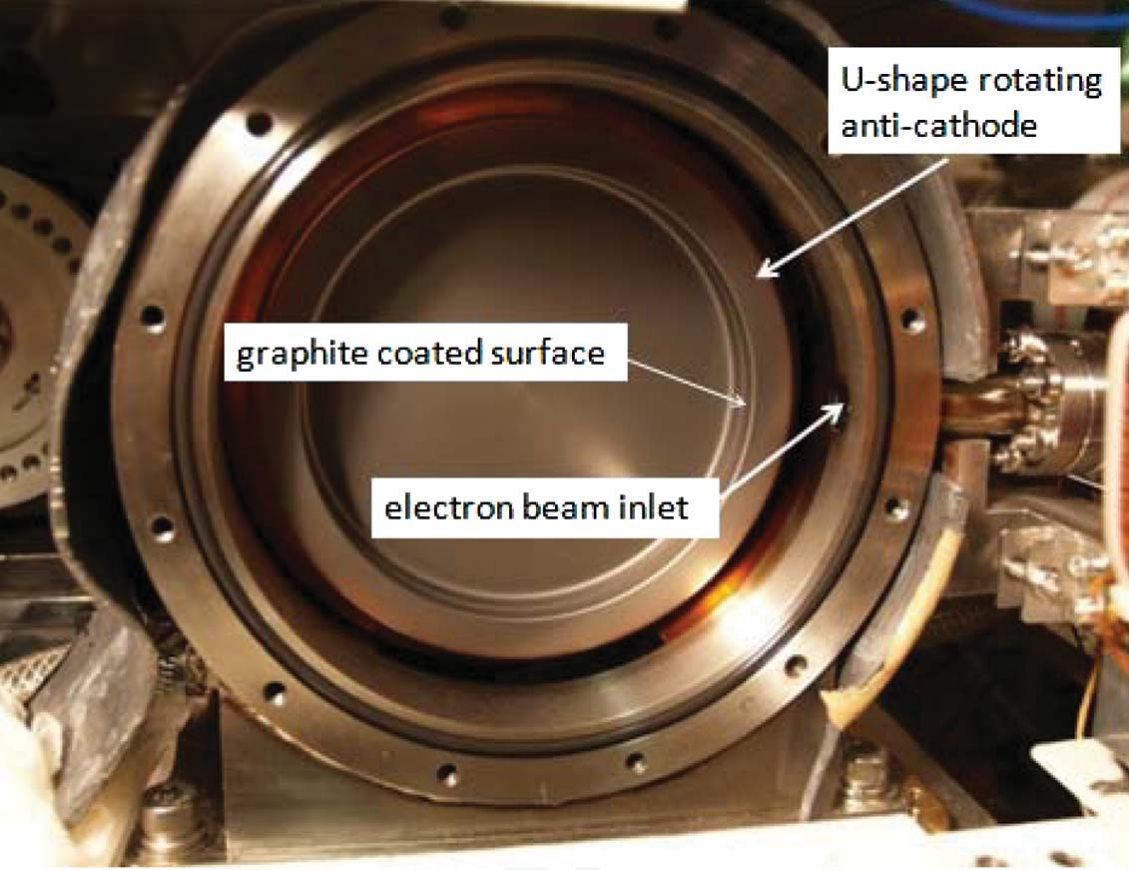
U-shape rotating anti-cathode after graphite coating.

Fig. 4 ▶ shows the U-shape rotating anti-cathode whose target was coated with the thin carbon film. This rotating anti-cathode has been used for a total operation time of 181 h before the photograph of Fig. 4 ▶ was taken, and the irradiated part on the cylindrical inner surface was roughened by the thermal stress due to a high-power electron beam. This roughened area is seen in Fig. 4 ▶ as a light-colored narrow line in the middle part of the cylindrical target surface.

The mean roughness of this part is approximately at the 30 µm level. Coating with carbon film could not prevent the surface from this roughening.

### 3.3. Experiment with the coated target

#### 3.3.1. Temperature

The graphite-coated target withstood electron beam irradiation for 15.1 h and 28.9 h at 2130 K and 1890 K, respectively. Since the Cu target had been coated with graphite, all the temperature measurements shown below are not for Cu but for graphite. The output image, for example, of the two-color-type thermometer (commercial model Thermera-seen D414) is shown in the supplementary material (Fig. S1)^1^, when the beam voltage, current and focus area were 60 kV, 72 mA and 0.041 mm^2^, respectively. The small right-hand bottom frame in Fig. S1 shows a CCD thermal image of the irradiated target surface in which a length of only about 4 mm is observed as the part keeping a high temperature. The length of 4 mm corresponds to 0.127 ms in time length for the moving target surface.

#### 3.3.2. Brightness

**Figure 5:**
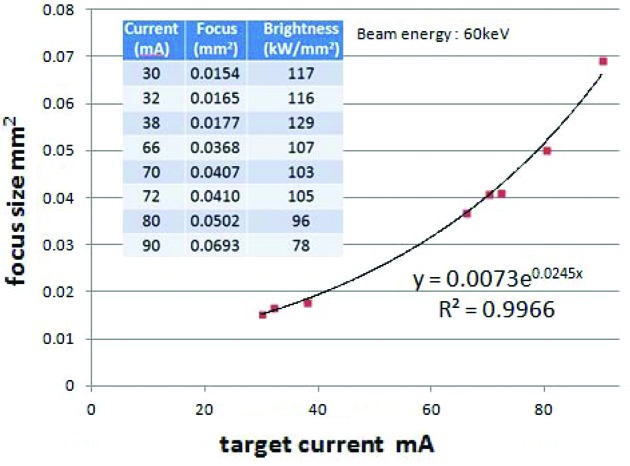
Brightness values of the U-shape target are ten times greater than those before modification and whose specific brightness was 12 kW mm^−2^.

Some examples of the experimental results are shown in the tabulated data in Fig. 5 ▶. The maximum brightness of 129 kW mm^−2^ was obtained with the parameters 60 kV × 38 mA, that is 2.28 kW, with the electron beam size being 0.0177 mm^2^ which corresponds to an ellipsoid whose minor and major axis sizes are 0.0525 mm and 0.430 mm, respectively. The reason that the maximum brightness was obtained at a rather low current is that the focus size is strongly dependent to the current. The tabulated data in the figure are accompanied by a graph of experimentally obtained focus areas *versus* beam current.

#### 3.3.3. Comparison between vertical incidence and oblique incidence

The trace of the electron beam on the surface of the target is roughened by thermal stress and the roughness amounts to about 30 µm. It was very difficult to remove the roughness by melting and the strong centrifugal force on our present generator. It was determined that the X-ray output decreased along with the development of the roughness in the original design of our generator.

This difficult problem has been solved by changing the condition of the electron beam incidence onto the target surface from being a vertical to an oblique incidence. To carry out experiments on the beam incidence conditions and the target surface conditions using an electron beam of up to 120 keV, a new DC power supply (DF120N9X4138 Spellman) was used. The CCD X-ray detector (Fig. 1 ▶) was also replaced by an S-SDD (X-tec) energy-dispersive X-ray detector to collect Cu *K*α X-rays. In order to keep the counting losses below 2%, the target current was kept below 0.5–1 mA. The voltage between the cathode and the anode was changed from 35 kV to 110 kV with an increment of 5 kV. The X-ray output intensity photons were collected through a 10 µm-diameter pinhole at a distance of 75 mm from the irradiated point as shown in Fig. 1 ▶. At the focus position, its size and shape were adjusted by watching the transition of light by using a simple CCD camera mode of the Thermera-seen D414.

The X-ray energy spectral counts between 7.7 and 8.3 keV were collected using the S-SDD detector.

**Figure 6:**
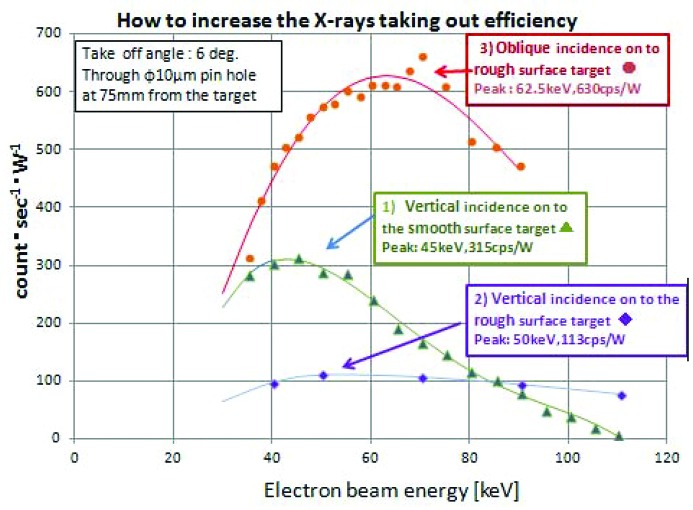
Observations of the X-ray intensity change of the U-shape target under three different conditions. The dimensions of counts s^−1^ W^−1^ can be changed to the brightness expression of photons s^−1^ mm^−2^ mrad^−2^ (0.1% bandwidth)^−1^ W^−1^ by multiplication by the coefficient constant 0.36 × 10^5^; and where the focus size was *S* = 1.96 × 10^−3^, the pinhole area was *s* = 78.54 µm^2^, and the pinhole distance from the focus point *L* = 75 mm, namely, *s*/*L* ^2^ = 1.4 × 10^−2^ mrad^2^ (0.1% bandwidth) being that of the Cu *K*α band pass.

Fig. 6 ▶ shows the Cu *K*α counts (1) at a vertical incidence onto an optically smooth surface new target, (2) onto the roughened surface caused by exposure to a high-power electron beam, and (3) at an oblique incidence onto a roughened surface target. The maximum counts are 315, 113 and 630 counts s^−1^ W^−1^ for (1), (2) and (3), respectively. The latter (630 counts s^−1^ W^−1^) was twice as large as the first one (315 counts s^−1^ W^−1^). This means that the efficiency of the X-rays extracted out *via* the Be window was larger by a factor of two compared with that with the vertical incidence onto the smooth surface.

### 3.4. Endurance evaluation with a limited operation time

Two U-shape targets have been produced. The first one was used for about two years before coating with graphite film and for about three years after coating. Then it was replaced by a new one to carry out the above-mentioned experiment (§3.3.3). The first one is still usable.

**Figure 7:**
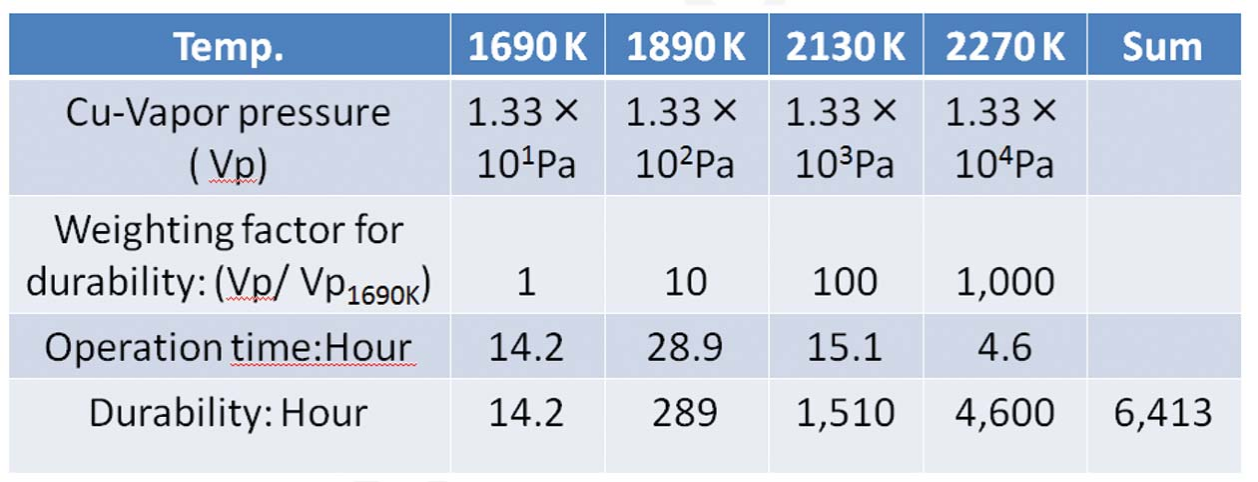
Experimental results of the endurance tests and thereby the estimation of the durability. Since the target is still usable, the U-shape target withstands more than 6413 h when it is used at 1690 K, which is over 334 K more than its melting point.

Endurance estimation was made using the first U-shape target with a graphite film coating and the results are shown in Fig. 7 ▶. The weighting factor for durability in Fig. 7 ▶ is determined by assuming that the lifetime of the target is inversely proportional to the target vapor pressure which depends on the temperature at the irradiating point of the target.

Considering the fact that the target is still usable, Fig. 7 ▶ suggests a durability lifetime of more than 6413 h if the target was used at a temperature of 1690 K, 334 K higher than the melting point of copper. This means that not only the specific heat but also the heat of fusion enables a high X-ray flux to be achieved.

## 4. Discussion

### 4.1. Why the oblique incidence is better than the vertical incidence

**Figure 8:**
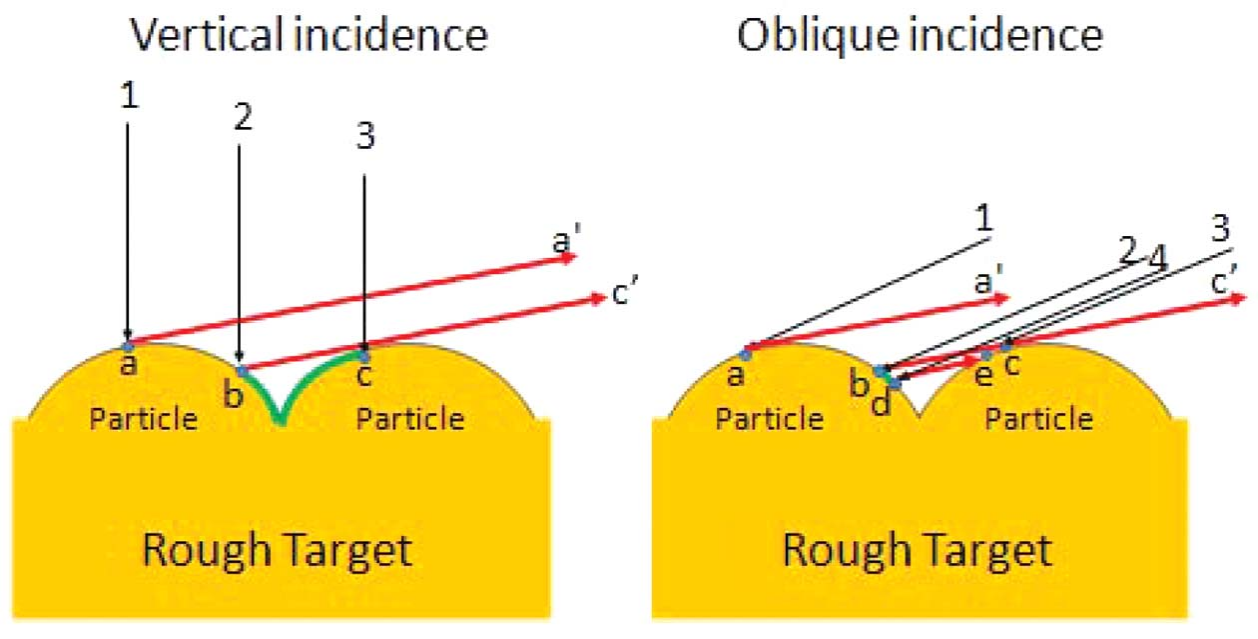
Schematic diagram showing the rough target surface to explain the difference between vertical incidence and oblique incidence geometries and their respective performances. Black arrows: electron beams. Red arrows: X-ray beams.

Fig. 8 ▶ shows a comparison of schematic geometrical situations between the vertical and oblique incidence conditions. Points *a* and *c* on neighboring particles are defined as those where the directions of the tangents on the particle surfaces (*a*–*a*′ and *c*–*c*′) are parallel to the X-ray exit direction. Point *b* is defined as the intersection of the tangent *c*–*c*′ and the surface of the left-hand-side particle. Arrows 1, 2 and 3 in both illustrations correspond to electron beams incident on the points *a*, *b* and *c*, respectively. Two more points, *d* and *e*, are shown in the illustration for oblique incidence. At point *e*, the incident beam, arrow 4, just contacts the particle as a tangent. The point *d* is the intersection of this tangent and the left-hand-side particle.

In the case of the vertical incidence, all the beams in the region between arrows 1 and 3 reach the surface from *a* to *c*. Among them, however, those in the region between 2 and 3 cannot contribute to the X-ray output in the exit direction because of interception by the right-hand-side particle. Also in the case of the oblique incidence, beams between 2 and 4 cannot contribute to the X-ray output in the exit direction similarly as in the former case. However, those between 4 and 3 can contribute to the X-ray output. This portion of beams appears by bringing the incidence angle closer to the exit angle. We consider the electron beams coming on to a particular area of the target surface from *a* to *c*, in other words, the total beams in the region between arrows 1 and 3. Among them the portion between 2 and 4 cannot contribute to the X-ray output in the exit direction. This portion would vanish if we could bring the incidence direction to coincide with the exit direction. This means all the incident beams would contribute to the X-ray output making the incident beam usage maximum. In other words, every beam reaches a point on the surface that can be seen from the exit direction at this incidence condition. It is expected that the usage factor of the electron beams will be improved by bringing the incident direction sufficiently close to the exit direction, though perfect coincidence of the two directions is impossible now because of the present location of the bending magnet. At its simplest we can say that the beam width between arrows 1 and 2 in the vertical incident generator is comparable with that in the oblique incident generator. The beam between arrows 2 and 3 could not contribute to the X-ray output. The beam width in the oblique incident generator is significantly narrower as compared with that in the vertical incident generator. Thus the oblique incident system decreases the loss of the X-ray output. Therefore the overall result is that the oblique incident system is superior to the vertical incident system.

## 5. Conclusion

Overall the X-ray brightness of the new U-shape-type X-ray generator has amounted to a level 20 times stronger than that of a conventional present state-of-the-art X-ray generator currently offered commercially.

## Supplementary Material

Supplementary material file. DOI: 10.1107/S0909049513022188/ys5092sup1.pdf

## Footnotes

1 Supplementary data for this paper are available from the IUCr electronic archives (Reference: YS5092). Services for accessing these data are described at the back of the journal.
