# Supplementary material for: U-shape rotating anti-cathode compact X-ray generator: 20 times stronger than the commercially available X-ray source
Source: J Synchrotron Radiat. 2013 Oct 2;20(Pt 6):829–33. doi: 10.1107/S0909049513022188 (PMC3795538; doi:10.1107/S0909049513022188)
Supplement: Supplementary file 1 [file s-20-00829-sup1.pdf]

U-shape rotating anti-cathode compact X-ray generator: 20 times stronger than the commercially available X-ray source

N. Sakabe[a,b], K. Sakabe[a,b], S. Ohsawa[c], T. Sakai[c], H. Kobayakawa[c], T. Sugimura[c] , M. Ikeda[c] ,M. Tawada[c], N. Watanabe[d], K.Sasaki[e], M.Wakatsuki [f] .

[a]Photon Factory, High Energy Accelerator Research Organization (KEK), 1-1 Oho, Tsukuba, Ibaraki 305-0801, Japan,
[b]Foundation for Advancement of International Science (FAIS), 16-24, kasuga 3-chome, Tsukuba, Ibaraki 305-0821, Japan,
[c]Accelerator Laboratory, High Energy Accelerator Research organization (KEK), 1-1 Oho, Tsukuba, Ibaraki 305-0801, Japan.
[d]Synchrotron Radiation Research Center, Nagoya University, Chikusa, Nagoya, Aichi 464-8603, Japan.
[e] Nagoya University, Chikusa, Nagoya, Aichi 464-8603, Japan.
[f] National Institute of Advanced Industrial Science and Technology (AIST), 1-1-1Higashi, Tsukuba, Ibaraki 305-8568.
E-mail: sakabe-dsb@sbsp.jp

Supplementary technical details

The irradiated spot consists of an ellipse with main axes, x and y, respectively along the rotation axis of the target and the direction of rotational movement of the target surface, respectively. The values of x and y, were determined as FWHM (Full Width Half Maxima) from the X-ray source image, as Dx=0.068mm and Dy=0.080mm. Since the take off angle was 6 degrees, the size of the electron beam along the rotation axis (δ_2) was 0.65mm (δ_2= 0.068/sin6°) while that along the rotational direction, δ_1 was 0.08mm. Thus, the area of the ellipsoid irradiated by the electron beam was 0.04mm^2 and the brightness at this stage was 66.1kW/mm^2 which is 5.5 times greater than 12kW/mm^2, that of the unmodified (commercial) X-ray generator.

Note that these reference values were already given in Sakabe N., et al.(2008)

JSR 15, 231-234.

So, area of which is simply calculated with δ1 x δ2 just like a tungsten filament

coil cathode. But this time we consider that we use the circular LaB_6 cathode, and thus we use the ellipsoid irradiated area, namely, π(δ1/2)(δ2/2) and reach "66.1 kW/mm^2 ie which is 5.5 times greater than 12 kW/mm^2" as quoted in the article abstract.

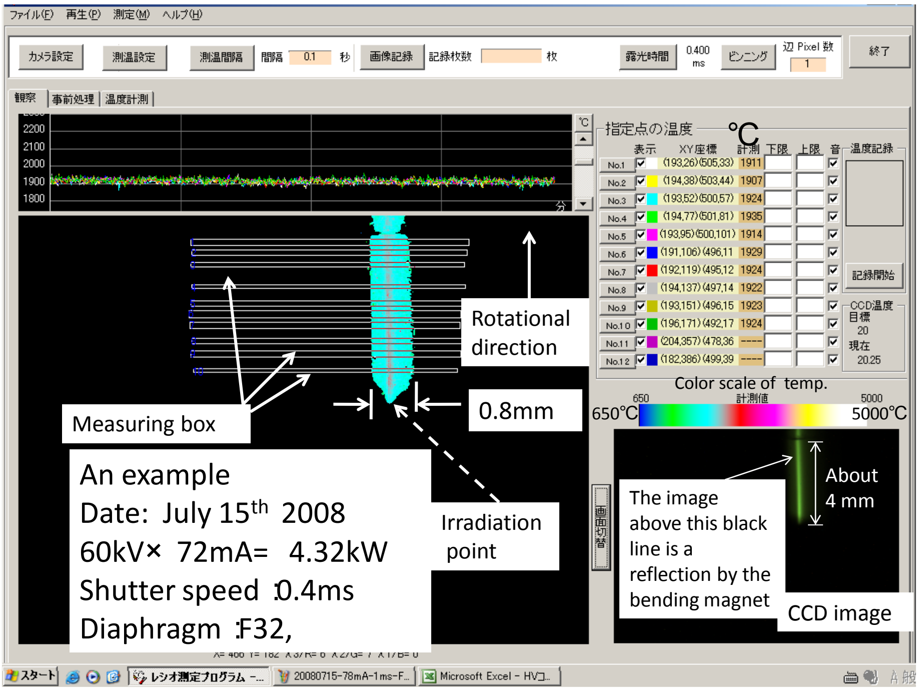

Figure S1.Target temperature of irradiation area was 2,100-2,400K. Further details to main text:-

The mean value for the measuring box used was about 1900 degrees C (~2200K). Near the center line of the green color band it shows slightly pink which means the high (and highest) temperature (see the color scale of the temperature). The optical lens used was a Thermera-seen. As the temperature was so high, the diaphragm of the lens used must be F32, and also the shutter speed (sampling time) had to be 0.40ms, otherwise it was too bright to measure.

Text accompanying Figure 5:-

Three series of data were taken under three conditions as specified in Figure 5 and whose details are given here.

1) Optically smooth target: The U-shape anti-cathode was replaced with a new, identically shaped, one. The bending magnet was not changed. This was the experimental condition for “Vertical incidence on to the smooth surface target”.

2) Roughened surface target: Before data taking the X-ray generator had been operated with a high power to keep the target temperature at about 2,000K for 20hours as a “roughening” treatment. No other conditions were changed. This was the experimental condition for “Vertical incidence on to the roughened surface target”.

3) Oblique incidence to the roughened target: A bending-magnet was newly designed for 45 degrees oblique incidence, and only the magnet poles were replaced with newly designed ones. This was the experimental condition for “ Oblique incidence on to the roughened surface target”.
